# Role of immunoglobulins in neonatal sepsis

**Published:** 2014-12-19

**Authors:** L Capasso, AC Borrelli, J Cerullo, R Pisanti, C Figliuolo, F Izzo, M Paccone, T Ferrara, S Lama, F Raimondi

**Affiliations:** Division of Neonatology, Section of Pediatrics, Department of Translational Medical Science, University Federico II Naples, Italy, (letizia.capasso@gmail.com)

**Keywords:** *neonate*, *immunoglobulins*, *sepsis*

## Abstract

**Neonates, especially VLBW, are at high risk for sepsis related morbidity and mortality for immaturity of their immune system and invasive NICU practices. The paucity of immunoglobulins in preterm neonates consequently to the immaturity of immune system contributes to their high risk for systemic infection. The use of intravenous IgM enriched immunoglobulins, with higher antimicrobial activity than standard IgG, has been demonstrated in a retrospective study to reduce short term mortality in VLBW infant with proven sepsis. Larger, randomized prospective trials given the enormous burden of morbidity and mortality imposed by neonatal sepsis should urgently be addressed not only to validate this results but also to tailor the optimal scheme of treatment.**

## I. INTRODUCTION

Neonatal sepsis still represents one of the main causes of mortality and morbidity among preterm especially very low birth weight (VLBW) infants [1–2]. Mortality in VLBW with septicemia can reach 50% [3]. VLBW infants are prone to infections because of immaturity of their immune system, immunoglobulins and complement deficiencies, invasive practices in NICU and lack of maternal transplacental transport of IgG that usually occurs after 32 wk of gestation [3–5]. Antibiotic treatment also immediately started, sometimes failed to prevent death; administration of intravenous immunoglobulins in such neonates could improve immunitary functions [6].

The fetus is in a condition of isolation and protection from the contact with microorganisms potentially responsible for infection, and has a deeply immature immune system. Several components of the immune system appear early during gestation however they become completely functioning long after birth [7] and in straight relation to the increasing gestational age. Consequently, the newborn and the infant have a significant immunodeficiency and are exposed to the risk of serious infections.

The reduced inflammatory response that characterizes the newborn also explains the lack of clinical signs of localization, such as fever or meningism, which often makes difficult to identify a systemic infection [8].

## II. INFECTION SUSCEPTIBILITY IN THE NEWBORN

There are several factors that increase the susceptibility to infection in the newborn, especially if preterm and very low birth weight infant, such as immunological deficiencies, need for invasive, diagnostic and therapeutic procedures.

A crucial role is played by the immaturity of the skin barrier. The skin of these infants is, in fact, immature and fragile, acting as an inadequate protective barrier against pathogens. Although skin rapidly matures after birth, its barrier function is compromised for more than 4 weeks; During this period, the infant experienced an excessive loss of transepidermic water [9], which is usually limited by applying 80% humidity in incubator, however this step at the same time promotes bacterial and fungal growth and colonization of the skin. Also respiratory and gastrointestinal mucosal appear immature and colonized by microorganisms that may have access through nasogastric and endotracheal cannulas normally used in such infants [10]. This occurs especially in relation to the deficit of secretory class A immunoglobulin, mucins and defensins, important in ensuring adequate mucosal defense. Also intestinal peristalsis and absorptive capacity are compromised and levels of intraepitelial lymphocytes are reduced [11].

Innate immunity in the newborn, compared to the adult, is also impaired in numerous functions. Neutrophils and monocytes have less capacity of migrate to the inflammation site [7]; this occurs because the chemotactic activity, as well as the random migration, is inadequate. Phagocytosis and microbicidal activity are, however, similar to adult subjects [12,13]. The cytokines production, in particular IL-6 [14] and TNF-α [15], are compromised, and this justifies the reduced febrile response of the newborn. Although the percentage of NK cells in fetal blood is lower compared to the child or the adult, the absolute number is almost the same; however they have a decreased activity, in particular in the response to signaling [16]. The APC (antigens presenting cells), especially dendritic cells, also present a reduced function [17,18]; it would explain the impairment of T-mediated response in fetus and newborn [19,20].

Non-specific humoral factors have lower serum concentrations than in adult. This deficit is the more pronounced the smaller the gestational age is. The fetal synthesis of complement proteins begins from the 5th – 6th month of intrauterine life [12]; there is no evidence of transplacentar transport [7, 21–23]. The levels of the various fractions are lower [7]; it depends on a lack of self-synthesis and is inversely related to gestational age. In term newborn activation of the classical pathway is altered in association to the opsonizing immunoglobulins deficiency while the alternative pathway acquires a compensatory role [12]; in preterm they are both compromised. This deficit, in association to the reduction of phagocytic activity can increase the possibility of acquiring serious infections, particularly from extracellular pathogens [19]. The right balance between pro-and anti-inflammatory cytokines appears to be essential for the elimination of the pathogen and the resolution of the same inflammatory state with minimal residual damage. When this does not occur both morbidity and mortality are markedly increased [24].

Fetal thymus is a lymphoid organ active after the sixth week of gestation, characterized by an intense lymphopoiesis independent by the antigenic stimulus [25]. The production of T lymphocytes increases gradually during the second trimester, reaching normal values within the 30°–32° week, however, these cells are functionally inadequate: CD4 + T cells are able to generate clonal expansion, but are not sufficiently competent in helper function and the cytotoxic activity is considerably reduced [8].

Another very important aspect is the deficiency of MHC class II molecules, a condition that persists for a long time and is correlated with increased susceptibility to infections. However it is necessary, in order to maintain the state of immunological tolerance, especially in early pregnancy, so that the product of conception is not recognized as foreign by the mother and then rejected [12]. B lymphocytes appear in fetal bone marrow, blood, liver and spleen before the 12th week of gestation, from this period begins the active placental transfer of maternal antibodies [26]. Only IgG can cross the placental barrier because of their low molecular weight and the presence of specific receptors on the face of the placental chorionic villi. This allows the achievement in term newborn of IgG levels equal or even higher than the adult subject. In preterm infants, however, the antibody levels are much lower, the lower the gestational age is [12]. Therefore preterm [26] and VLBW infant [27] can present a significant hypogammaglobulinemia that inevitably exposes them to a greater risk of infection. At birth, most of immunoglobulins are maternal IgG, whose concentration decreases rapidly as a result of physiological catabolism and whose specificity is strongly dependent on the experience of maternal antigen.

Infant antibody production begins around the 3rd month of life and by the age of 4–6 years IgG levels reach adult levels. The fetus is not able to produce IgA, therefore at birth mucous membranes are totally devoid of antibody defense and more susceptible to infections. However, colostrum, rich in secretory IgA, allows a valid compensation [12]. Newborn has an increased susceptibility to Gram-negative pathogens infection in relation to the absence of maternal IgM transport, which act as heat-stable opsonins, and the low levels of C3b, heat-labile opsonin, that correlates with the impaired phagocytic activity of polymorphonuclears. Maternal IgG can adequately compensate the function of heat-labile opsonin and provide protection against many Gram-negative pathogens, but not in the preterm infant that has deficient levels of immunoglobulins [16].

## III. IMMUNOGLOBULINS AS ADJUVANT TREATMENT IN SEPSIS

Intravenous immunoglobulins supplementation could represent an appealing strategy to fight neonatal sepsis especially in preterm and VLBW infant.

Administration of intravenous immunoglobulins in such neonates could improve immunitary functions such as opsonisation, complement activity, antibody dependent-citotoxicity, and neutrophil chemoluminescence [28]. Several authors studied the role of immunoglobulins in the treatment of neonatal sepsis [29–31]. In 2010, a Cochrane metanalysis has demonstrated that intravenous immunoglobulins administration significantly reduce mortality in neonates with suspected or proven infection [32]. Recently, the INIS study, an international, placebo-controlled, multi-centre randomised trial that has enrolled 3493 infants and tested the adding of standard immunoglobulins (SIVIG) to antibiotic therapy in neonates with suspected infection showed that SIVIG had no effect on death or major disability at the age of 2 years [33].

In theory, IgM-enriched IVIG (IgM-IVIG) have a higher antimicrobial activity than S-IVIG because of higher opsonization activity and activation of complement than IgG. A commercial formulation of IgM-IVIG, containing a high titre of IgM, IgA and IgG against both Gram− and Gram+ organisms [29] showed in animal and human studies that in models of Gram-bacteremia increases oxidative burst, reduces endothelial damage and liver and spleen colonization [34]. In adult human patients with severe sepsis or septic shock a significant reduction in mortality as well as a significant decrease in a severity illness score (APACHE score) were found [35]; the IgM-enriched IVIG administration in combination with antibiotics showed an improvement of the survival of surgical ICU patients with abdominal sepsis [36] A reduction of procalcitonin levels and days with fever in patients with post-surgical infections treated with IgM-enriched IVIG were found [37]. A cost-effectiveness analysis also demonstrated that such product is a promising adjuvant therapy both clinically and economically for treatment of adults with severe sepsis and septic shock [38]. Few studies investigated the role of IgM-IVIG in neonates: Haque et al compared the use of S-IVIG and IgM-IVIG and found a significant reduction of mortality in neonates treated with IgM-IVIG compared with those treated with S-IVIG and controls with a faster normalization of laboratory parameters in IgM-IVIG group [29]. The same authors in a previous study demonstrated a reduction of mortality in neonates (GA 28–37 wk) with suspected sepsis treated with IgM-IVIG versus placebo [39]. There are no studies focused on the use of IgM-enriched IVIG in the treatment of sepsis in very low birth weight (VLBW) infants.

## IV. USE OF IGM IVIG AS ADJUVANT TREATMENT IN SEPSIS OF VLBW INFANTS

Our group conducted a retrospective, cohort study on the use of IgM-IVIG in addition to antibiotic therapy in VLBW neonates with late onset sepsis as an hypothesis generator for future prospective clinical trials [40].

All VLBW infants enrolled were included in the local section of the Vermont Oxford Network (VON) database from January 2008 to December 2012 for a total of 459 neonates reviewed. Neonates were born at the University “Federico II” of Naples, the largest delivery place in the Naples regional area assisted by a level III NICU. Inclusion criteria was the diagnosis of blood culture-proven, late onset sepsis (i.e. sepsis occurring after 72 hours of life) in VLBW infants. For defining blood culture as positive, we adopted the Vermont Oxford Network criteria, i.e.:

- sepsis by coagulase negative staphylococcus: pathogen recovered from either a central line, or peripheral blood sample in association to one or more signs of generalized infection and treatment with 5 or more days of intravenous antibiotics after the above cultures were obtained;
- sepsis by other bacteria: bacterial pathogen recovered from blood culture;
- sepsis by fungi: fungus recovered from a blood culture obtained from either a central line or peripheral blood sample [41].

Clinical signs for the diagnosis were : apnoea, mottled skin, temperature instability, feeding intolerance, significant abdominal distension, respiratory distress or hemodynamic instability). Laboratory index used were elevated CRP (cut off =1 mg/dL), abnormal leukocyte count (cut off less than 5.000/µl or more than 20.000/µl) and I/T ratio (cut off >0.2). To assess the clinical severity of cases and controls at enrollment, we used the SNAP II score, a composite index of six physical parameters (hypotension, hypothermia, acidosis, PO_2_/ FiO_2_ ratio, multiple seizures, urinary output) initially designed for NICU admission [42]. A recent study validated the SNAP II score as a accurate mortality predictor at the onset of severe neonatal sepsis [43]. For this study, SNAP II score was calculated for each patients used the parameters found in the chart in the first 24 hours sepsis was suspected (i.e. when the clinical deterioration was reported, the blood exams were performed included blood culture and antibiotics were started).

Exclusion criteria were: congenital anomalies, TORCH group infections, primary immunodeficits. Intravenous antibacterial therapy used throughout the study was teicoplanin (loading dose 16 mg/Kg followed by 8 mg/Kg q 24 hours) and meropenem(20 mg/Kg q 8 hours). In the event of a positive blood culture for Candida spp, antibiotic therapy was withdrawn and a standard antifungal regimen (liposomal amphotericin B: 5 mg/kg/die) was used for 3 weeks.

Starting June 1st 2010, IgM-IVIG (Pentaglobin® Biotest Pharma, Dreieich, Germany) 250 mg/kg/die i.v. for three days was added to the NICU protocol for sepsis treatment in the first 24 hours sepsis was suspected when antibiotic therapy was started. In order to compare neonates before and after the introduction of IgM-eIVIG in our clinical practice, meticulous care was exerted to ascertain that no other major change had been made that could have been relevant to the study outcome (i.e. central lines managing and duration; fluconazole prophylaxis against invasive fungal infections; handwashing and other general prophylactic measures; enteral nutrition).

VLBW preterms with late onset sepsis may have a prolonged hospital stay after a septic episode and their demise might be related to many factors other than infection; therefore, short-term mortality (i.e. death within 7 and 21 days from treatment) was an appropriate primary outcome for this study. Secondary outcome measures were: in-hospital total mortality, rates of intraventricular hemorrhage, periventricular leukomalacia, necrotizing enterocolitis, bronchopulmonary dysplasia at discharge.

**Figure 1.**
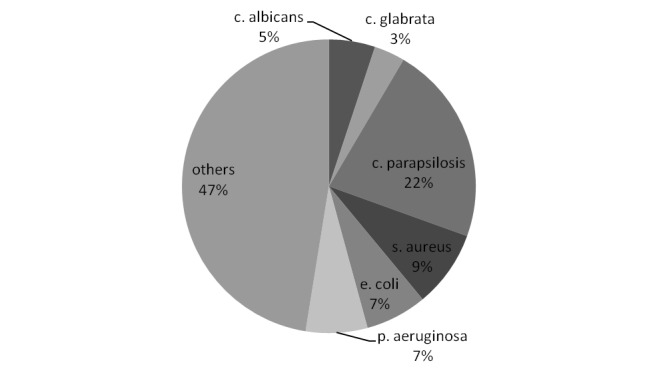
Main pathogens isolated from blood cultures

Of the 82 VLBW infants enrolled, 2 neonates were excluded for congenital anomalies and one for TORCH infection; of the remaining 79 (see demographics in Table 1), 40 patients received antibiotics in association to IgM-IVIG and 39 received antibiotic treatment alone. No difference in birth weight, gestational age or SNAP II score was found. All enrolled infants had a positive blood culture and the microbiology is given in figure 1.

Mortality at 7 and 21 days coincided. IgMIVIG treated infants had a significantly lower short term mortality than untreated (OR 0.16; 95% CI: 0.3–0.7, p=0.005). Secondary outcomes were not significantly different between cases and controls (table 2).

In a subgroup analysis of neonates with Candida spp. sepsis, fewer short term deaths were also found for immunoglobulin treated neonates compared with untreated neonates. In fact 1/10 (10%) neonates died in treated group versus 8/15 (53%) deaths in untreated group (OR 0.1; 95% CI: 0.01–0.97, p=0.047).

## V. CONSIDERATIONS ON THE USE OF IGM IVIG AS ADJUVANT TREATMENT FOR SEPSIS IN VLBW INFANTS

This pilot study shows that IgM-IVIG are effective in reducing short-term mortality in VLBW infants with proven sepsis. When compared with S-IVIG, IgM-IVIG show more efficient complement activation, better opsonisation, greater neutralization of the streptococcal superantigen SpeA and better binding both to bacterial antigens and toxins all possibly due to their pentameric structure [44]. This biological background together with our retrospective observation may increase the interest towards this strategy for IgM-IVIG in VLBW infants with sepsis despite the paucity and somewhat conflicting specific clinical evidence in the literature.

With a bigger cohort we detect a clinically relevant difference focusing only on the most susceptible babies. Under the standard conditions of VON, we show an effect of IgM-IVIG on short term mortality on those babies who had sepsis proved by positive blood culture.

Unlike the Haque series, our most prevalent pathogens were fungi against which IgM-IVIG proved to be very effective. We later found out that our study preparation had an average anti-C. albicans specific titer of 1.618 U/ml of IgG, 171 U/ml of IgA, 194 U/ml of IgM (courtesy of the manifacturer).

The explanation for this novel observation is possibly related to the fact that antibodies are a dominant protective mechanism against disseminated candidiasis.

In fact, antibody opsonization is crucial to optimal Candida phagocyting and killing by neutrophils and monocytes. Moreover, antibodies against Candida mannan antigens activate complement [45].

Despite the encouraging results, our study finds the main limitation in its observational nature; however, searching for confounders, we were not able to detect significant change of NICU practices or outcome variations in the study period. Also, mortality is an immediate clear-cut endpoint.

We believe that this analysis fulfilled its original purpose to set the ground for larger, randomized prospective trials. Given the enormous burden of morbidity and mortality imposed by neonatal sepsis, new research should urgently be addressed not only to validate our results but also to tailor the optimal scheme of treatment.

## Figures and Tables

**TABLE 1.** MAIN CHARACTERISTICS OF THE WHOLE STUDY POPULATION

	TREATED	UNTREATED	
	n=40	n=39	p
Gestational Age (weeks)	27±2,6	27,6±3,9	NS
Birth Weight (grams)	924±277	951±362	NS
Snap II Score	15±13	12±9	NS
Cesarean Section	39 (59%)	38 (58%)	NS
Prenatal Steroids	32 (48%)	39 (60%)	NS
CRP Positive	51 (77%)	49 (75%)	NS

**TABLE 2.** PRIMARY AND SECONDARY OUTCOMES OF THE WHOLE STUDY POPULATION

	TREATED	UNTREATED	OR (95%CI)	p
	n = 40	n = 39		
Short term mortality	9 (22%)	18 (46%)	**0.16 (0.3–0.7)**	0,005^*^
Total mortality	17 (44%)	18 (46%)	0.46 (0.9–1.8)	NS
IVH	9 (22%)	8 (20%)	1.17 (0.5–2.7)	NS
PVL	3 (7,5%)	2 (3 %)	2.6 (0.5–14)	NS
NEC	2 (6%)	1 (3%)	2 (0.4–11)	NS
BDP	4(10%)	4 (10%)	0.97 (0.2–4.2)	NS
